# Precision Nutrition to Improve Risk Factors of Obesity and Type 2 Diabetes

**DOI:** 10.1007/s13668-023-00491-y

**Published:** 2023-08-23

**Authors:** Janet Antwi

**Affiliations:** Department of Agriculture, Nutrition and Human Ecology, Prairie View A&M University, Prairie View, USA

**Keywords:** Precision nutrition, Personalized nutrition, Nutrigenetics, Metabolomics, Metagenomics, Risk factors, Behavior change, Dietary patterns, Obesity, Type 2 diabetes

## Abstract

**Purpose of Review:**

Existing dietary and lifestyle interventions and recommendations, to improve the risk factors of obesity and type 2 diabetes with the target to mitigate this double global epidemic, have produced inconsistent results due to interpersonal variabilities in response to these conventional approaches, and inaccuracies in dietary assessment methods. Precision nutrition, an emerging strategy, tailors an individual’s key characteristics such as diet, phenotype, genotype, metabolic biomarkers, and gut microbiome for personalized dietary recommendations to optimize dietary response and health. Precision nutrition is suggested to be an alternative and potentially more effective strategy to improve dietary intake and prevention of obesity and chronic diseases. The purpose of this narrative review is to synthesize the current research and examine the state of the science regarding the effect of precision nutrition in improving the risk factors of obesity and type 2 diabetes.

**Recent Findings:**

The results of the research review indicate to a large extent significant evidence supporting the effectiveness of precision nutrition in improving the risk factors of obesity and type 2 diabetes. Deeper insights and further rigorous research into the diet-phenotype-genotype and interactions of other components of precision nutrition may enable this innovative approach to be adapted in health care and public health to the special needs of individuals.

**Summary:**

Precision nutrition provides the strategy to make individualized dietary recommendations by integrating genetic, phenotypic, nutritional, lifestyle, medical, social, and other pertinent characteristics about individuals, as a means to address the challenges of generalized dietary recommendations. The evidence presented in this review shows that precision nutrition markedly improves risk factors of obesity and type 2 diabetes, particularly behavior change.

## Introduction

Obesity and diabetes have emerged as enormous public health problems not only in the USA but also globally. Diabetes is a significant global challenge to the health and well-being of individuals and societies [1]. With a continued global increase in diabetes, the current prevalence of 537 million adults living with diabetes is projected to rise to 643 million by 2030 [1]. In the USA, an estimated 37.3 million people have diabetes, of which 90–95% of cases, including children, adolescents, and young adults are attributed to type 2 diabetes [2–4]. Diabetes data and trends for 2019 available at the Centers for Disease Control and Prevention indicated that diabetes is the sixth leading cause of death, and number one cause of kidney failure and lower limb amputation [3, 4]. Obesity is the strongest risk factor for the development of type 2 diabetes [5–7]. Thus, the burden of type 2 diabetes is increasing in parallel to increasing cases of obesity [8]. Clinical data show that of the people diagnosed with type 2 diabetes, about 80–90% are highly likely to be diagnosed as obese [9–12]. The associated medical expenses of obesity and type 2 diabetes are steep. Obesity costs the US health care system nearly $173 billion a year [13, 14], while the total estimated economic burden of type 2 diabetes was $327 billion in medical costs and lost productivity [15].

Both obesity and type 2 diabetes have related multifactorial etiology, making them highly complex diseases and investment in their effective prevention and management has become necessary to tackle this global epidemic. While obesity and type 2 diabetes have traditionally been studied to be diseases of energy imbalance, other risk factors such as high body weight and fat, dyslipidemia, high blood glucose, and insulin resistance are also involved in the etiology [16–18]. Unhealthy diet characterized by foods high in fat, sugars, and calories, but low in plant-based sources, and lack of physical activity are now considered top risk factors for the development and progression of obesity and type 2 diabetes [19]. Thus, improving dietary intake and physical activity is a global priority [20].

Dietary recommendations and public health campaigns for tackling risk factors of obesity and type 2 diabetes have focused on using population averages, have been based on generalized advice, or have been poorly adhered to [21–24]. Moreover, there have been great challenges with the validity, consistency, and reproducibility of dietary assessments [25]. Because obesity and type 2 diabetes are heterogeneous diseases from the pathophysiological, genetic, and clinical perspectives, and there is dramatic inter-individual variability in response to any therapeutic diet or physical activity regime, there is a need to shift to or complement the population perspective with patient-centric interventions [26–28]. These variabilities are attributed to differences in genetics, biomarkers of metabolic pathways, gut microbiome, environmental, physiological, behavioral, social, and economic factors. Given the substantial burden of obesity and its related comorbidities, research and practice efforts should adopt a holistic approach for sustainable solutions in preventing and treating the obesity and type 2 diabetes epidemic [9].

**Fig. 1:**
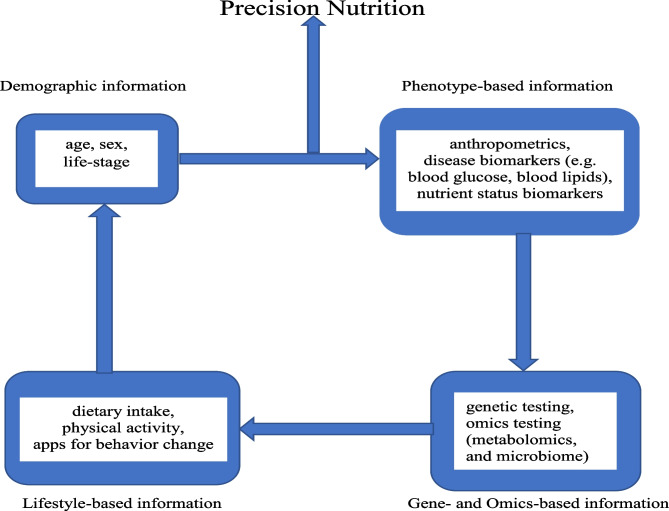
Components of the precision nutrition approach. The individual characteristics of demographic, phenotype, lifestyle, genetic, and omics information are incorporated into the precision nutrition intervention to address the interpersonal variabilities in response to general nutrition intervention and recommendations to improve the risk factors of obesity and type 2 diabetes

Precision nutrition (or personalized nutrition) has emerged as a new area of lifestyle intervention that allows dietary recommendations to be tailored at the individual level through integration of demographic information, lifestyle-based information (e.g., dietary intake, and physical activity), phenotype-based information (e.g., anthropometrics, and standard clinical biomarkers of disease risk), and gene- and omics-based information (e.g., genetic testing of single nucleotide polymorphisms, and gut microbiome) (Fig. 1) [29, 30]. The current use of nutrigenetics, metabolomics, and metagenomics in precision nutrition enables the holistic interrogation of dietary and lifestyle factors to objectively assess risk factors of obesity and type 2 diabetes. The identification of various genes and polymorphisms has been determined as the basis for the interpersonal variability in metabolic response to specific diets [31–33]. Metabolomics investigates, among other things, the effect of food-derived biomarkers metabotypes variation among individuals in metabolizing the same diets in health and disease states for customized dietary interventions through metabolic patterns [34]. The identification of metabolites of food intake to serve as target of nutrition intervention makes metabolomics have potential to improve the accuracy of dietary assessment [35]. Metagenomics is vital in precision nutrition because it can be used to comprehensively analyze the diet-microbiome interaction to identify various metabotypes that characterize metabolic risk and tailor dietary intervention approaches for improved health [36].

It is suggested that precision nutrition interventions could result in greater weight loss and blood glucose control than non-personalized strategies [37, 38]. In personalizing nutritional advice, there is evidence that people are more motivated to make appropriate behavioral changes [39, 40]. The interest in precision nutrition has not only significantly increased in the scientific community [41], but is already becoming more accessible to consumers, largely through self-administered test-kits coupled with diet plans and subscription programs [41–43]. Thus, precision nutrition has been identified as the individualized solution to prevent and manage obesity and type 2 diabetes in lieu of the population-based dietary interventions, whose effectiveness in reducing the risks of these conditions using the “one-way diet” approach for all individuals is questionable [44].

The purpose of this review is to examine the current state of the science regarding precision nutrition in improving the risk factors of obesity and type 2 diabetes with emphasis on studies that included more than one component of precision nutrition and not only genetic testing to provide individualized/personalized dietary advice. While progress has been made on the quantity of research focused on precision nutrition, reviews discussing particularly behavior change and changes in nutrient/diet quality and physical activity as part of a comprehensive analysis of the utility of precision nutrition intervention and its outcomes are lacking.

## Nutrigenetics

Nutrigenetics is considered the foundation of precision nutrition (Table 1) [45, 46]. Genetic variation in the form of single nucleotide polymorphisms (SNPs) is considered to account for the heterogeneity in individual dietary response and risk for obesity and type 2 diabetes [47, 48]. Nutrigenetic research has investigated the interactions between SNPs influencing body composition, insulin signaling, and dietary factors in relation to adiposity and glucose homeostasis in obesity and type 2 diabetes. In an observational study, a genetic risk score-diet interaction used to provide precision nutrition based on 16 SNPs related to obesity or lipid metabolism demonstrated its value in obesity prediction. Specifically, in individuals carrying > 7 risk alleles, there was higher body mass index (BMI), body fat mass, waist circumference, and waist-to-hip ratio more than the individuals with ≤ 7 risk alleles [49]. Additionally, there was a significant interaction between genetic risk score and the macronutrient intake used in personalized intervention. Similarly, a systematic review and meta-analyses and two observational studies reported genetic interactions with specific macronutrients, that is, carbohydrate [50], fat [51], and protein intakes, respectively [52]. SNPs in the apolipoprotein A1 and C3 (*APOA1 and APOC3*) genes and cluster of differentiation 36 (*CD36*) gene led to increased risk of metabolic syndrome in subjects with Western dietary pattern and dyslipidemia in individuals who consumed high amounts of fat, respectively. Two randomized controlled trials (RCT) showed that personalized prescription of energy-restricted diets (low-fat and moderately high-protein) based on 95 different genetic variants related to energy homeostasis, phenotypic, and environmental factors was associated with differential adiposity outcomes, with waist circumference and total body fat loss particularly among obese subjects who carried the Peroxisome Proliferator Activator Receptor Gamma Coactivator 1 (*PPARGC1A* Gly482Gly) genotype [53••, 54]. In an observational prospective cohort design from the RCT, Prevención con Dieta Mediterránea (PREDIMED), the investigators concluded that genetic predisposition to type 2 diabetes associated with the Transcription Factor 7-Like 2 Gene [*TCF7L2* gene (rs790314 TT)] homozygosity could be counteracted through precision nutrition interventions with the Mediterranean diet [55]. While precision nutrition effectively addresses the genetic variability in nutrient metabolism, and other physiological processes among individuals, it was found in a parallel-group, pragmatic, RCT that providing nutrigenetic information and advice for management could help reduce body fat percentage up to 6 months, and reductions in body fat were similar to the standard weight loss intervention after 12 months. The clinical implications of this study are that the genetic-based precision nutrition approach should be considered for use for clinical cases which require short- to long-term body fat loss, particularly for individuals needing that to undergo surgery or transplant [56]. The Preventing Overweight Using Novel Dietary Strategies (POUNDS LOST) RCT was conducted to determine the impact of precision nutrition on fasting glucose, fasting insulin, hemoglobin A1C (HbA1C), insulin resistance, and β cell function. The precision nutrition diet varied in macronutrient composition and was investigated with type 2 diabetes genetic risk scores on these parameters of glucose metabolism. At 2 years of intervention, low-protein diet responses significantly interacted with lower genetic risk score and greater decreases in fasting insulin, HbA1C, insulin resistance, and a lesser increase in β cell function, compared to those with a higher genetic risk score [57]. A post hoc analysis of the POUNDS LOST RCT showed that in response to high-fat diets, participants with the highest genetic risk score showed increased fasting glucose, insulin resistance, and decreased insulin sensitivity at 6-month follow-up than those with low-fat diets [58]. The influence of genetic factors and nutrient-gene interactions in precision nutrition applications has been indicated by twin studies. In the Personalized Responses to Dietary Composition Trial (PREDICT) RCT [59••], a large inter-individual variability in postprandial blood glucose and insulin responses was observed following the same meals among 1002 twins and unrelated healthy adults in the UK. Genetic variants had modest impact on predictions of glucose, triglycerides, and C-peptide. These results were independently validated among 100 US adults. In addition, a machine learning algorithm predicted these variabilities to precision nutrition. An observational retrospective pre/post comparison of digital twin-enabled precision nutrition therapy was used to examine diabetes reversal [60••]. The authors reported diabetes reversal (that is, achieving HbA1C < 6.5% at least 3 months after stopping antidiabetic medications) during 90 days of precision nutrition therapy at varying rates of subgroups of obese and non-obese type 2 diabetes patients. Baseline data showed that only 9.5% of patients were in reversal stage 4 or better; however, over the first 90 days, 82.1% achieved advanced stages of reversal with improved clinical outcomes and fewer pharmacotherapy. Furthermore, a retrospective study reported that there was a decrease in HbA1C, body weight, fasting blood glucose, and insulin resistance at 90-day follow-up assessment [61]. In contrast, a prospective RCT [62] that randomized overweight or obese individuals to receive a nutrigenetic-based precision nutrition diet or standard balanced diet reported no difference in weight loss between the two groups. However, the results highlight the need for larger macronutrient differences between groups and adherence to the recommended intervention diet plan. Further research should be conducted to provide new data and make the use of genetic-based precision nutrition management in the clinical setting more effective [62]. Studies on diet-gene interactions among non-Caucasians are limited. In a prospective cohort study of Hispanics of Caribbean origin who were genotyped for the *Perilipin* SNP [*PLIN 11482G* > *A* (rs894160)] to determine whether dietary macronutrients modulated the associations of the SNP with obesity (measured as BMI, waist and hip circumference), the investigators found that the minor allele was protective against obesity for subjects who consumed higher complex carbohydrate, whereas among those with lower complex carbohydrate intake, the minor allele was linked with increased risk of obesity [63].

**Table 1:** Summary of evidence supporting utility of precision nutrition approaches in improving risk factors of obesity and type 2 diabetes

**Authors, year (reference)**	**Participants (*n*; age; country; health)**	**Study design; duration; intervention**	**Risks measured**	**Relevant findings**
**Nutrigenetics**				
Ramos-Lopez et al. [53••]	305; > 18 years; Spain; overweight/obese adults	RCT; 4mo; 2 hypocaloric diets (30% restriction) with different macronutrient distribution: a low-fat diet (22% energy from lipids) and a moderately high-protein diet (30% energy from proteins)	Anthropometrics, blood pressure, lipid profile, dietary intake and physical activity, SNPs	Greater fat loss among obese subjects who carried the *PPARGC1A* Gly482Gly genotype
Corella et al. [55]	7018; 55–80y; Spain; healthy, and adults with type 2 diabetes	Observational prospective cohort design; 9y; One of 3 diets: a Mediterranean diet supplemented with extra-virgin olive oil, a Mediterranean diet supplemented with mixed nuts, or a control diet (advice to reduce dietary fat)	Anthropometrics, fasting glucose, lipid profile, SNPs, new onset of type 2 diabetes	Following a Mediterranean diet was associated with reduced genetic predisposition to type 2 diabetes associated with the *TCF7L2* gene (rs790314 TT) homozygotes
Huang et al. [57]	744; > 18 years; USA; overweight or obese nondiabetic adults	RCT; 2 y; 4 diets differing in macronutrient composition: moderate in fat (40%) with two different protein levels (15% and 25%), and low in fat (20%), also with 15% and 25% protein levels.	Anthropometrics, fasting glucose, insulin, β cell function, SNPs	Low-protein weight-loss diet improved insulin resistance and β cell function in individuals with lower genetic risk of diabetes, whereas a high-protein diet was beneficial for a higher genetic risk.
Berry et al. [59••]	1002; 18–65y; UK; healthy adults	Intervention clinical trial; clinic visit and 13 days at home; postprandial metabolic responses to sequential mixed-nutrient dietary challenges	Fasting glucose, lipid profile, gut microbiome profile, SNPs	Genetic variants had a modest impact on predictions (9.5% for glucose, 0.8% for triglyceride, 0.2% for c-peptide)
Shamanna et al. [60••]	463; > 18 years; India; type 2 diabetes patients	Observational retrospective pre/post comparison; 90 days; machine learning algorithms analyzed patient’s macronutrients, micronutrients, and biota nutrients	Anthropometrics, HbA1c; lipid profile	Diabetes reversal, and a decrease in HbA1C, body weight, fasting blood glucose, and insulin resistance
Frankwich et al. [62]	51; > 18 years; USA; overweight or obese adults	RCT; 8–24 weeks; nutrigenetic-guided diet (balanced, low-carbohydrate, low-fat, or Mediterranean) or a standard balanced diet	Body weight, SNPs	Nutrigenetic-based diet did not increase weight loss compared with a standard balanced diet. Genetic features could identify beneficial strategy
Smith et al. [63]	920; 45–74y; USA; health adults	Cohort study; 2y; dietary macronutrients (e.g. carbohydrates and fats)	Anthropometrics, SNPs	Minor allele of *PLIN 11482G* > A (rs894160), protective against obesity in higher complex carbohydrate group
**Metabolomics**				
Anwar et al. [68•]	40; 28–65y; Canada; healthy adults	Cohort study; 100 days; web-based personalized lifestyle recommendations, including diet, exercise, and nutritional supplement	Metabolite biomarkers	Reductions in risks of development of type 2 diabetes, insulin resistance and related comorbidities
Bouwman et al. [70]	33; > 18 years; Netherlands; healthy adults	Controlled cross-over study, 5 weeks, response groups to dietary intervention with anti-inflammatory ingredients	145 metabolites, 79 proteins	Nutritional intervention reduction on oxidative stress, inflammation and metabolism differed between treated and untreated
Fiamoncini et al. [71]	70; 55–63y; UK; healthy adults	RCT; 12 weeks; standardized mixed meal tolerance test	Body composition, insulin level, fasting glucose, lipid profile, metabolites	Only individuals with higher disease-linked metabotype demonstrated improvements in glucose and insulin levels when fed a low caloric diet.
Zheng et al. [72]	1092; 42–60y; USA; overweight and obese adults	RCT; 2y; dietary intervention	Body weight, insulin resistance, plasma amino acid metabolites	Personalized weight-loss diets decreased circulating amino acid metabolites that were associated with risk of type 2 diabetes, and improved insulin resistance, independent of weight loss
Walford et al. [73]	757; 40–60y; USA; adults with prediabetes	Nested case–control; 2y; intensive lifestyle intervention focusing on a healthy diet and exercise	Anthropometrics, fasting glucose, fasting insulin, 84 metabolite biomarkers	Dietary and lifestyle modifications increased betaine which effectively predicted lower risk of type 2 diabetes
**Metagenomics**				
Zeevi et al. [82]	800 (initial), 100 (validation); 18–70y; Israel, USA; healthy adults	Cohort study and RCT; 1 week; precision nutrition designed with machine learning algorithm	Postprandial glucose, gut microbiome	Significantly lower postprandial glucose levels and consistent alterations in gut microbiome
Mendes-Soares et al. [83, 84]	327; 33–57y; USA; adults without diabetes	Cohort study; 6 days; predictive model of individualized PPGRs to a diverse array of foods was trained and applied	Anthropometrics; postprandial glucose; gut microbiome;	A personalized predictive model was more predictive than current models of carbohydrate content
Kovatcheva- Datchary et al. [85]	39; > 18 years; Sweden; healthy adults	Cross-over study; 3 days; dietary intervention with barley kernel bread diet or white wheat flour bread	Blood glucose, gut microbiome,	Improved postprandial glucose metabolism was in those with statistically significant higher ratio of *Prevotella/Bacteroides* spp.
Fragiadakis et al. [87]	49; 18–50y; USA; healthy adults	RCT; 12mo; dietary intervention of low-carb or low-fat diet	Body weight, gut microbiome	Baseline gut microbiota composition was not associated with weight loss, however there was substantial changes in gut microbiota in response to each diet, specifically low-carbohydrate at 3 months
Vangay et al. [88]	514; > 18 years; USA, Thailand immigrants; healthy adults	Cross-sectional and longitudinal sub-study; one-time point, and 6mo; dietary acculturation	Gut microbiome	U.S. immigration rapidly depleted gut microbiota diversity and function and was replaced by US-associated strains and functions, and was exacerbated by obesity.
Yu et al. [89]	144; > 18 years; China; healthy adults	Cohort study; 3y; long-term healthy or unhealthy diet, determined by a healthy diet score	Body weight, gut microbiome	Long-term healthy diet intervention was associated with greater diversity of *Tenericutes*, *Firmicutes*, *Actinobacteria*, with or without adjustment for BMI
Cuevas-Sierra et al. [90••]	190; 18–67y; Spanish; overweight and obese adults	RCT integrative gut microbiota-genetic model of a moderately high protein diet, and a low fat diet; 4mo	Anthropometrics, lipid profile, insulin, gut microbiome, SNPs	Mixed models microbiota scores facilitated the selection of diet in 84% of men and 72% of women for weight loss
**Behavioral (dietary patterns, and physical activity) aspects of precision nutrition**				
Hietaranta-Luoma et al. [112]	107; 20–67y; Finland; healthy adults	RCT; 1y; behavioral effects assessed for three groups: a high-risk, a low-risk, and a control group	Behavior changes measured by dietary intake, physical activity, alcohol consumption, apoE genotypes	Personal genetic information affected health behavior. Dietary fat quality improved more in the high risk group than in the control groups
Nielsen & Sohemy [113]	138; 23–35y; Canada; healthy adults	RCT; 12mo; nutrition-related genetic information for personalized nutrition on dietary intakes of caffeine, vitamin C, added sugars, and sodium	Dietary intake	Greater reduction in sodium intake for DNA-based dietary advice than general population-based recommendations
Celis-Morales et al. [114]	1488; 18–79y; 7 European countries; healthy adults	RCT, 6mo; Dietary advice based on current diet; Dietary advice based on current diet and phenotype (anthropometry, blood markers); Dietary advice based on current diet, phenotype, and genotype	Anthropometrics, dietary intake, biomarkers	Precision nutrition group consumed less red meat, salt, and saturated fat, increased folate intake and had higher HEI scores
Livingstone et al. [115]	1480; 18–79y; 7 European countries; healthy adults	RCT; 6mo; Dietary advice based on current diet; Dietary advice based on current diet and phenotype (anthropometry, blood markers); Dietary advice based on current diet, phenotype, and genotype	Dietary intake	Precision nutrition enhanced dietary behavior changes associated with higher Mediterranean-style diet scores than in controls
Horne et al. [133]	140; 40–68y; Canada; overweight and obese adults	RCT; 12mo; genotype-based advice for macronutrient recommendations using Theory of Planned Behavior	Dietary intake and dietary adherence	A nutrigenomics weight-management intervention can motivate greater long-term dietary change compared with general advice
Marsaux et al. [137]	1607; 18–79y; 7 European countries; healthy adults	RCT; 3–6mo; 3 level internet-based personalized advice: on current physical activity plus diet; physical activity plus diet; and phenotype, or physical activity plus diet, phenotype, and genotype	Physical activity (objective and self-reported)	Self-report based physical activity levels increased to a greater extent with more personalized nutrition advice, but not when physical activity was objectively measured
Zhou et al. [138]	64; 40–52y; USA; healthy adults	RCT; 10 weeks; automated machine learning mobile phone-based personalized and adaptive goal-setting intervention	Physical activity	Personalized and adaptive goal-setting intervention promoted behavior change in physical activity
Godino et al. [139]	569; 39–55y; UK; healthy adults	RCT; 2mo; Physical activity advice through phenotypic-based type 2 diabetes risk or genotype-based type 2 diabetes risk	Physical activity, body weight, dietary intake, health-related behavior and attitudes, SNPs	No observation of changes in physical activity behavior after communicating genetic or phenotypic estimates of type 2 diabetes
Nielsen et al. [140]	1002; > 18 years; USA; healthy adults	Cohort study; 6mo; personal genomic testing intervention on health behaviors	Dietary intake, physical activity	Personal genomic testing significantly increased strength exercise frequency which was attributed to direct motivation of personal genetic testing results to make behavior changes

*RCT *randomized controlled trial, *PPARGC1A Gly482Gly *peroxisome proliferator activator receptor gamma coactivator 1, *TCF7L2 gene (rs790314 TT)* transcription factor 7-Like 2 gene, *HbA1c *hemoglobin A1c, *SNPs *single nucleotide polymorphisms, *PLIN 11482G* > *A (rs894160) *Perilipin SNP, *HEI *Healthy Eating Index, *PPGRs *postprandial glycemic responses

## Metabolomics

Metabolomics, an emerging technology which encompasses comprehensive analysis of metabolites, holds promise to inform precision nutrition recommendations (Table 1) [64]. The various metabolites produced from metabolism of dietary factors have been used to characterize metabolic phenotypes or biomarkers that can be used for individual stratification. This metabolic specificity enables precision nutrition to resolve metabolic derangements that underlie obesity and type 2 diabetes [34]. Additionally, metabotyping which stratifies individuals with metabolic similarity into metabotype subgroups using their metabolic and phenotype patterns could be used for population stratification to customize dietary interventions [65]. Earlier studies that paved the way for the use of metabolomics in precision nutrition showed that dietary intake patterns were revealed in metabolomic profiles [66], and were associated with biomarkers such as high levels of lipid metabolites, amino acids, and ferritin that mediated red meat consumption and risk of type 2 diabetes [67]. Recently, a study analyzed blood metabolites using metabolomics among normoglycemic healthy adults to predict the risk of developing type 2 diabetes. A web-based platform interventional study was used to deliver precision nutrition intervention based on the blood metabolites health risk score to lower the blood metabolites to normal levels for 40 participants. A follow-up assessment of the blood metabolites showed significant reductions in the health risks associated with the development of type 2 diabetes, insulin resistance, and related comorbidities [68•]. A replication of the study through observational longitudinal analysis in a larger cohort of 1000 US adults demonstrated similar positive results with the precision nutrition intervention given based on biomarkers measured through metabolomics [69]. Bouwman et al. [70] in a double-blind placebo-controlled cross-over design used a health space model to visualize the effect of personalized nutrition intervention on metabolic stress profile including inflammatory and oxidative processes associated with obesity and type 2 diabetes. After following the recommendations for 5 weeks, the 145 metabolites and 79 proteins measured prior and before treatment were able to distinguish modulation of metabolic stress and specific oxidative and inflammatory response to treatment. Fiamoncini et al. [71] in an experimental design identified 2 metabotype clusters and tested their responses to a personalized nutrition intervention over a 12-week weight loss program. The researchers reported that only the study participants with higher disease-linked metabotype demonstrated improvements in glucose and insulin levels when fed a low caloric diet. They concluded that through the application of metabolomics in precision nutrition advice, a responsive and non-responsive metabotype was revealed. In the DIRECT (Dietary Intervention Randomized Controlled Trial) trial, personalized weight-loss diets decreased circulating amino acid metabolites that were associated with risk of type 2 diabetes, and improved insulin resistance. In addition, the reduction in the level of circulating amino acid metabolites which is indicative of an increase in insulin sensitivity was independent of weight loss [72]. Walford and colleagues performed plasma metabolite profiling to elucidate new pathways of type 2 diabetes incidence and the role of personalized nutrition interventions in a nested case–control design [73]. Dietary and lifestyle modifications based on the metabolites effectively raised betaine concentration from baseline to 2-year follow-up, which predicted lower risk of type 2 diabetes. Interestingly, a 10-week RCT that allocated 100 overweight and obese adults to a personalized diet and control diet based on their metabolomic and genetic information did not show significant difference between groups in fat mass; however, the individual diets produced significant improvements in insulin resistance and lipid profile, which was not significantly different between groups. The soundness of various precision nutrition approaches is required to translate such findings into clinical relevance [74•].

## Metagenomics

Metagenomics is the comprehensive study of host microbial and their genetic material (Table 1) [75]. The role of the gut microbiota in obesity and type 2 diabetes has been underscored, and this has been an area of immense research [76]. It is believed that the metabolism of dietary compounds into other metabolites by the gut microbiota, which is associated with disease risk, mediates the impact of the gut microbiota on human health [77–79]. For example, the metabolism of dietary fibers and resistant starches into bacterial metabolites of short-chain fatty acids such as acetate, propionate, and butyrate presents a mechanism that modulates the pathways involved in obesity, insulin resistance, and type 2 diabetes [80]. Studies show that the diet-gut microbiota interactions vary in composition and functionality among individuals [81], and this appears to be a determinant to integrate metagenomics into precision nutrition [36]. Pioneering work by Zeevi et al. [82] in an observational study and blinded randomized controlled dietary intervention showed that postprandial glucose responses have high interpersonal variability even when individuals consumed identical standardized diets. The authors further used a machine learning algorithm that integrated dietary habits, blood parameters, anthropometrics, physical activity, and gut microbiota features for precision nutrition recommendations in the 800 person cohort. The precision nutrition recommendations accurately predicted personalized postprandial glucose response to the recommendations and resulted in significantly lower glucose levels and consistent alterations in gut microbiome. In modifying and extending the model created by Zeevi and colleagues, two cohort studies that evaluated the utility of such precision nutrition approaches to predict postprandial glucose responses found that across the cohort of non-diabetic adults that were examined, a personalized model was more predictive than current models of carbohydrate content [83, 84]. Similarly, Kovatcheva-Datchary et al. [85] in a cross-over study demonstrated that among 39 healthy Swedes, improved postprandial glucose metabolism was in those with statistically significant higher ratio of *Prevotella/Bacteroides* spp., following an intervention of 3-day consumption of barley kernel bread diet. Another RCT demonstrated through metagenomic analysis and a dietary weight loss intervention that compared to individuals with a low bacterial ratio, subjects with a high *Prevotella/Bacteroides* genera ratio lost more weight and body fat in response to high-fiber diets [86]. In a sub-study of a larger RCT, researchers examined whether the baseline composition and diversity of gut microbiota was associated with weight loss in a sample of 49 participants. Findings from the study showed that baseline gut microbiota composition was not associated with weight loss; however, there were substantial changes in gut microbiota in response to each diet, 3 months after initiating the intervention. The changes were attributed specifically to the healthy low-carbohydrate diet used in the intervention, although the changes were attenuated after 12 months [87]. Another important step in the use of metagenomics in precision nutrition was the work conducted by Vangay et al. [88] in an observational study that provided valuable insight into differences in population groups that requires racial considerations and sociocultural influences when employing precision nutrition approaches. In this study, Karen and Hmong natives residing in Thailand and the USA as well as European Americans born in the USA were assessed for the impact of migration to the USA on the gut microbiota in development of metabolic diseases such as obesity. After metagenomic DNA sequencing, the investigators found that US immigration rapidly depleted gut microbiota diversity and function and was replaced by US-associated strains and functions, and was exacerbated by obesity. These results were confirmed in a prospective cohort study that used similar metagenomic approaches of 16S and deep shotgun DNA sequencing among 144 Chinese individuals in Shanghai. A long-term healthy diet intervention was associated with greater diversity of *Tenericutes*, *Firmicutes*, and *Actinobacteria*, with or without adjustment for BMI [89]. Data from an RCT of an integrative model using gut microbiota and genetic information to personalize weight loss prescription among 190 Spanish overweight and obese participants suggested that the mixed models’ microbiota scores facilitated the selection of the optimal diet in 84% of men and 72% of women for weight loss [90••].

## Behavioral (Dietary Patterns, and Physical Activity) Aspects of Precision Nutrition

Healthy behaviors (e.g., consuming a healthy diet and engaging in regular physical activity) are associated with the incidence of morbidity and mortality of chronic diseases including obesity and type 2 diabetes [91]. Behavior change components that may be beneficial to improve adoption of healthier options are goal setting, social interactions, and customized messages [92, 93]. Diet and physical activity behaviors are the strongest risk factors for obesity and type 2 diabetes prevention and outcomes [94]. Given this crucial role of behavior in preventing and treating chronic diseases, it is important to assess behavior change in dietary patterns and physical activity for improvement. The 2019 global burden of disease study reported that among the 3 largest increases in risk exposure for disability-adjusted life years (DALYs) lost across the world, 2 were high BMI and high fasting plasma glucose, and 6 of the top 10 causes of DALYs are due to poor health behaviors, including unhealthy dietary patterns and low physical activity levels [95]. Diet quality which represents the nutritional adequacy of a diet with varied nutrient composition, measured by how closely dietary patterns are within core nutrient-dense food groups, is a higher priority than the quantity of dietary intake [96–99]. In a systematic review of prospective cohort studies, a strong association was found between poor diet quality and greater weight gain, irrespective of gender [100]. In addition, higher diet quality is demonstrated in several studies to be associated with chronic disease risk, cause-specific mortality, and all-cause mortality [101–103]. Diet quality in the USA remains far from optimal and for all Americans, the average diet quality measured by the Healthy Eating Index (HEI) score is 58, which is far from the maximum of 100 points [104]. The top dietary risk factors in the USA are diets low in fruits, vegetables, whole grains, nuts, and legumes, and high in refined grains, red or processed meats, sodium, saturated and trans fats, and sugar-sweetened beverages [21, 105–107]. The transition from heavy labor to sedentary livelihoods, increased screen time, decrease in school physical education, and improved transportation has been implicated in the decline in physical activity levels [18, 107]. Studies show that moderate to vigorous-intensity physical activity such as walking or running is necessary for optimal health. A systematic review and meta-analysis of prospective cohort studies [108] reported that individuals who engaged in the minimum recommended amount of physical activity had potentially significant benefits to reduce the risk for type 2 diabetes by 26%, compared with inactive individuals. Thus, improvement in diet and physical activity signifies a huge potential for obesity and type 2 diabetes reduction either directly or indirectly through improvements in weight gain and blood glucose levels. It has been suggested that conventional dietary advice does not have as big of an impact on improving dietary health as expected [109, 110].

Precision nutrition interventions have demonstrated encouraging changes in dietary behaviors (Table 1). Precision nutrition studies that reported on behavior changes observed as healthy dietary patterns found that optimizing dietary patterns through individualized care improves management of obesity and type 2 diabetes [111–113]. For example, a randomized controlled trial that provided personalized nutrition advice using individualized information on diet and lifestyle, phenotype and/or genotype, produced larger, more appropriate, and sustained changes in dietary behavior to healthier diet as food groups compared to a conventional approach. Study participants in the precision nutrition group consumed less red meat, salt, and saturated fat, increased folate intake, and had higher HEI scores [114]. In line with these results, another RCT [115] that considered application of a dietary pattern technique instead of individual food items in isolation has reported that the use of precision nutrition enhanced dietary behavior changes associated with higher Mediterranean-style diet scores. The Mediterranean diet, characterized by high intakes of fruit and vegetables and low intakes of sugar-sweetened beverages and snacks, has been consistently linked with a beneficial effect on health, including obesity and type 2 diabetes [116–118]. Thus, it is strongly suggested that changing dietary intakes so as to align more appropriately with the Mediterranean diet would yield extensive public health benefit [119]. Through post hoc analyses, findings of the study further supported the importance of personalized nutritional advice which, when done with increased frequency, promoted sustained changes in dietary behavior and larger improvements in overall diet quality [120]. The changes in behavior of dietary patterns through the implementation of precision nutrition recommendations have also been associated with reduced intake of calories, carbohydrates, sugar, total fat, and saturated fat which correlated with significant weight loss, reduced waist circumference, and increased high density lipoprotein (HDL), decreased total cholesterol and low density lipoprotein (LDL) with improved glucose levels through observational studies, single-arm, multi-phase, open-label exploratory trial, and retrospective analysis of an RCT [121, 122••, 123, 124]. A pretest–posttest pilot study that organized a personalized dietary advice in a real-life setting found that dietary quality measured by the Dutch Healthy Diet Index was significantly improved compared with baseline. In addition, this research revealed that personalized dietary advice resulted in positive effects in self-perceived health in motivated pre-metabolic syndrome adults. Because the study was performed in the real-life setting (do-it-yourself), it highlighted the potential of at-home health behavior improvement through dietary changes [125]. The EatWellUK is another RCT that attests to the advancement of precision nutrition research beyond the USA. The authors of this research reported that an automated precision nutrition advice via a mobile web app was effective to elicit beneficial dietary change, improve diet quality, and increase engagement in healthy dietary behaviors in UK adults, relative to general population-based dietary guidelines [126••]. Similarly, other precision nutrition interventions found behavior change in dietary intake which favored healthier choices and increase in diet quality irrespective of the setting and/or platform used for delivery of the intervention, as well as measure used to assess diet quality score [127, 128••]. Short-term dietary behavior changes are usually very short lived, thus long-term compliance to dietary behavior change should not be compromised because it is crucial in maintaining body weight and blood glucose levels [129]. Generally, long-term dietary changes are difficult when it comes to consistency; however with the application of precision nutrition, there is a potential to optimize dietary behavior change by motivating greater adherence and change in dietary intake for the long-term for improved weight and glucose management [130–132]. The nutrigenomics overweight/obesity and weight management (NOW) trial was an RCT that shed more light on long-term dietary behavior change and adherence. More specifically, the investigators described that the use of precision nutrition increased motivation to long-term reduction in total fat intake, and long-term adherence to total fat and saturated fat advice [133].

Evidence shows that fixed step goals that are not personalized can discourage individuals, leading to unchanged behavior or even reduced physical activity levels [134–136]. There are findings, however, that show that the effect of precision nutrition to promote behavior change in physical inactivity and improve physical activity levels is not as consistent as observed for behavior changes in dietary patterns and diet quality. The findings of an RCT that included 1279 participants in 7 European countries to determine the effects of personalized advice on physical activity showed that while self-report-based physical activity levels increased to a greater extent with more personalized nutrition advice, there was no difference between the effect of personalized advice to promote changes in physical activity levels and conventional guidelines when physical activity was objectively measured. The authors concluded that it is vital to measure physical activity objectively in any physical activity intervention study [137]. Studies that analyzed objective measurement of physical activity levels in personalized advice support this theory as they found association between personalized and adaptive goal-setting intervention and steady daily steps, but not with constant steps in the control group, thus promoting behavior change in physical activity [138]. These data are in contrast with the results of an RCT that reported no changes in physical activity behavior after a precision nutrition intervention using objectively measured physical activity [139]. Nevertheless, an observational study found that precision nutrition significantly increased strength exercise frequency which was attributed to direct motivation of their personal genetic testing results to make behavior changes [140]. However, genetic results were not consistently associated with physical activity changes. Together these studies provide important insights into the precision nutrition effects on physical activity behavior changes, which highlights the need for further research.

## Conclusion

The current review provides evidence that although the application of precision nutrition is emerging, it is to a large extent associated with obesity and type 2 diabetes and may be effective approach in improving the risks factors including dietary patterns, physical activity, body weight and fat, blood lipids, blood glucose, and insulin resistance. This advancement has been enabled through the use of cutting-edge omics technologies which provide genetic, biomarkers, and microbiome insights into variabilities in individual metabolic pathways in response to dietary intakes that may impact health. It is worth noting as presented in this review that the evidence for precision nutrition is stronger for behavior change than for actual hard endpoints but maintaining the behavior changes in the long term is important for the hard endpoints to change, and this is challenging. The choosing of genetic and phenotypic parameters as a rational basis for individual-level, precision nutrition advice is a key factor that motivates people to make appropriate behavioral changes. However, individual health aspirations, food preferences, and barriers/facilitators to behavior change need to be considered and integrated more using a biopsychosocial model in developing precision nutrition approaches to maintain long-term behavior change and promote sustainability for better health outcomes [141]. In addition, there are still methodological challenges in the design and application of precision nutrition in clinical settings and scale up to the population level in addressing obesity and type 2 diabetes. While sensitivity and specificity issues of the omics technologies exist, some studies do not incorporate all the sources of individual variability in their assessment, and others do not have relevant behavior change techniques, are of short duration in their intervention, low diet quality, and of small sample sizes to observe an effect. More rigorous and well-executed RCTs are required to reinforce the evidence base for precision nutrition to be widely and effectively used in clinical setting and the public health domain. Moreover, increasing the reliability and reducing the cost of cutting-edge omics technologies and new frontiers in machine learning will undoubtedly pave the way for comprehensive and integrated framework of big data to combine multi-omics approaches with lifestyle and behavioral, phenotype, sociocultural, and demographic factors. This will help apprise the optimal design of precision nutrition interventions in clinical settings, and improve population diets at scale in improving the risk factors of obesity and type 2 diabetes. The vast majority of present knowledge and research on precision nutrition has been derived from developed countries [142]. It is crucial to conduct original research in other populations with different dietary habits, disease susceptibility, genetic makeup, socioeconomic characteristics, and health-related lifestyles. Extending precision nutrition research and application by examining and understanding a wider array of multi-race population health, technological and digital landscape, and political will are needed to ensure that there is equity prior to implementation of such approaches.
